# Optimizing electrical efficacy of leadless cardiac resynchronization therapy and leadless left ventricular septal pacing: Insights on left and right ventricular activation from electrocardiographic imaging

**DOI:** 10.1016/j.hroo.2024.07.004

**Published:** 2024-07-05

**Authors:** Nadeev Wijesuriya, Marina Strocchi, Mark Elliott, Vishal Mehta, Felicity De Vere, Sandra Howell, Nilanka Mannakkara, Baldeep S. Sidhu, Jane Kwan, Paolo Bosco, Steven A. Niederer, Christopher A. Rinaldi

**Affiliations:** ∗ King’s College London, London, United Kingdom; † Guy’s and St Thomas’ NHS Foundation Trust, London, United Kingdom; ‡ Imperial College London, London, United Kingdom; § Alan Turing Institute, London, United Kingdom

**Keywords:** Cardiac resynchronization therapy, Heart failure, Leadless pacing, Endocardial pacing, Electrocardiographic imaging

## Abstract

**Background:**

Leadless cardiac resynchronization therapy (CRT) is an emerging heart failure treatment. An implanted electrode delivers lateral or septal endocardial left ventricular (LV) pacing (LVP) upon detection of a right ventricular (RV) pacing stimulus from a coimplanted device, thus generating biventricular pacing (BiVP). Electrical efficacy data regarding this therapy, particularly leadless LV septal pacing (LVSP) for potential conduction system capture, are limited.

**Objectives:**

The purpose of this study was to evaluate the acute performance of leadless CRT using electrocardiographic imaging (ECGi) and assess the optimal pacing modality (OPM) of LVSP on the basis of RV and LV activation.

**Methods:**

Ten WiSE-CRT recipients underwent an ECGi study testing: RV pacing, BiVP, LVP only, and LVP with an optimized atrioventricular delay (LV-OPT). BiV, LV, and RV activation times (shortest time taken to activate 90% of the ventricles [BIVAT-90], shortest time taken to activate 95% of the LV, and shortest time taken to activate 90% of the RV) plus LV and BiV dyssynchrony index (standard deviation of LV activation times and standard deviation of all activation times) were calculated from reconstructed epicardial electrograms. The individual OPM yielding the greatest improvement from baseline was determined.

**Results:**

BiVP generated a 23.7% improvement in BiVAT-90 (*P* = .002). An improvement of 43.3% was observed at the OPM (*P* = .0001), primarily through reductions in shortest time taken to activate 90% of the RV. At the OPM, BiVAT-90 improved in patients with lateral (43.3%; *P* = .0001; n = 5) and septal (42.4%; *P* = .009; n = 5) LV implants. The OPM varied by individual. LVP and LV-OPT were mostly superior in patients with LVSP, and in those with sinus rhythm and left bundle branch block (n = 4).

**Conclusion:**

Leadless CRT significantly improves acute ECGi-derived activation and dyssynchrony metrics. Using an individualized OPM improves efficacy in selected patients. Effective LVSP is feasible, with fusion pacing at LV-OPT mitigating the potential deleterious effects on RV activation.

## Graphical abstract

**Figure:**
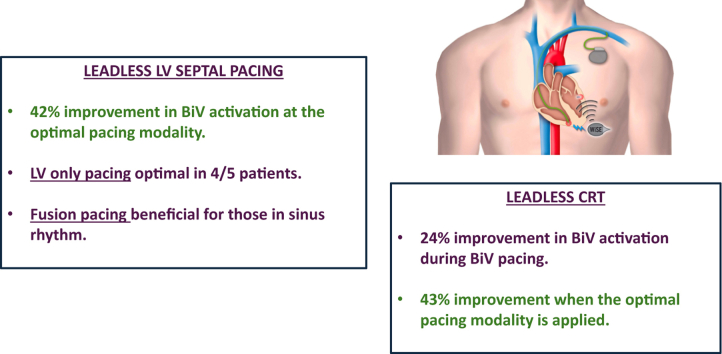

**Key Findings**

▪ Biventricular leadless endocardial cardiac resynchronization therapy (CRT) improves electrocardiographic imaging–derived electrical activation and dyssynchrony metrics.
▪ Biventricular (BiV) pacing may not be the *optimal pacing modality* in all patients. The optimal modality is dependent on patient factors, such as the underlying rhythm, and procedural factors, such as the location of left ventricular (LV) pacing.
▪ For those receiving leadless LV septal pacing, LV-only pacing may be superior to BiV pacing.
▪ For those with sinus rhythm and left bundle branch block, atrioventricular optimized LV-only pacing (fusion pacing) may be superior to BiV pacing.
▪ As such, individualized optimization of leadless CRT based on patient and procedural factors is likely necessary to maximize device performance and thus potentially benefit clinical outcomes.

## Introduction

Leadless left ventricular (LV) endocardial pacing, delivered by the WiSE-CRT system (EBR Systems Inc, Sunnyvale, CA), is an emerging form of cardiac resynchronization therapy (CRT)^1^ in patients with heart failure who are CRT-eligible^2^ who are unable to receive conventional transvenous LV epicardial pacing. The advent of conduction system pacing (CSP) gives operators a transvenous alternative in cases where conventional CRT is limited by unfavorable coronary sinus anatomy, LV lead displacements, or intolerable phrenic nerve stimulation postimplantation^3^ and in conventional CRT nonresponse.^4^ Nevertheless, leadless CRT is an important option in patients where implantation is restricted by absent venous access or prohibitively high infection risk and in those where CSP is also problematic, such as in congenital heart disease or in those with septal myocardial scar.

The system consists of 3 components: a subcutaneous battery, which is connected to an ultrasound transmitter placed in the fourth to sixth intercostal space, and a receiver electrode (RE) implanted in the LV cavity via femoral access. The patient requires a “coimplant” capable of right ventricular (RV) pacing. Upon detection of an RV pacing (RVP) impulse from the coimplanted device, the transmitter uses ultrasound pulses to communicate with the RE, converting ultrasound energy into an electrical pacing stimulus. This results in LV pacing (LVP) and thus near-simultaneous biventricular pacing (BiVP).

The efficacy of leadless CRT has been evaluated in observational studies.^1^^,^^5^, ^6^, ^7^, ^8^ A meta-analysis of these studies demonstrating a clinical response rate of 63% and an echocardiographic response rate of 54% at 6 months postimplantation,^9^ with favorable cost-effectiveness analysis also reported.^10^

The system was originally designed to deliver lateral wall LV endocardial pacing, with the RE implanted via retrograde femoral artery access. However, the development of a transseptal LV approach using antegrade femoral venous access^11^^,^^12^ permits LV septal RE deployment. While lateral LVP targeting the latest activating LV myocardium^13^ is a mainstay of conventional CRT, LV septal implantation may provide benefit via capture of His-Purkinje fibers, which run superficially in this region, to achieve leadless CSP.^14^ Reductions in QRS duration using BiVP with a septally implanted RE have been demonstrated.^14^

Currently, BiVP is the only clinically programmable setting for the WiSE-CRT system. However, BiVP may not be the optimal pacing modality (OPM), especially in patients with LV septal pacing in whom fusion with optimized atrioventricular (AV) delay could represent the optimal modality for LV and RV activation.^15^

We performed a mechanistic electrocardiographic imaging (ECGi) evaluation of leadless CRT to determine the acute efficacy of leadless CRT and leadless LV septal pacing on LV and RV activation metrics.

## Methods

### Study population

Ten patients from our center previously implanted with WiSE-CRT devices were included. This included all 5 patients with a septal RE, and 5 consecutive patients with a lateral RE attending the pacing clinic. All patients provided written informed consent. The study was conducted in accordance with the Declaration of Helsinki and approved by the local research ethics committee (13/LO/1475). The study results did not influence the subsequent patient care. After testing, devices were returned to baseline settings.

### ECGi study

Patients were fitted with a 252-electrode CardioInsight sensor array vest (Medtronic, Minneapolis, MN) and underwent a noncontrast computed tomography scan to obtain electrode positions and cardiac anatomy as previously described.^16^ Surface electrograms were continuously recorded throughout a noninvasive pacing protocol. Body surface potentials from individually selected beats were combined with the computed tomography–derived segmented cardiac anatomy to create reconstructed unipolar electrograms using the CardioInsight Workstation.

### Pacing protocol

A noninvasive acute pacing study was performed using the patients’ implanted devices (Online Supplemental Table S1). The following modalities were tested in this order: underlying rhythm, RVP, BiVP, LVP, and LVP at AV delays (AVDs) varying from 80 to 200 ms for patients in sinus rhythm (SR) to determine the optimal AVD (LV-OPT). Each ECGi recording was 1 minute in duration, and this was repeated 3 times per modality. There was a 30-second waiting period between recordings, during which time device settings were reverted to RVP for consistency.

### Activation map creation and calculation of ECGi-derived metrics

Custom in-house code was used to create activation maps and calculate ECGi-derived metrics as previously described.^17^^,^^18^ For each pacing configuration, the following metrics were calculated from an average of the 3 analyzed beats: (1) LV activation time (LVAT-95)—shortest time taken to activate 95% of the LV; (2) LV dyssynchrony index—standard deviation of LV activation times; (3) BiV activation time (BIVAT-90)—shortest time taken to activate 90% of the ventricles; (4) BiV dyssynchrony index—standard deviation of all activation times; and (5) RV activation time (RVAT-90)—shortest time taken to activate 90% of the RV. More detailed methodology is described in the Online Supplement, as well as ECG-based validation of the ECGi-derived metrics for this cohort (Online Supplemental Figure S1) and a summary of prior studies where ECGi maps were validated against invasive mapping data.

### Statistical analysis

Continuous variables were tested for normality using the Shapiro-Wilk test and are expressed as mean ± SD if normally distributed. Discrete variables are expressed as count (percentage). For each dyssynchrony metric, the calculated end point is expressed as percentage improvement compared to baseline. *Baseline* was defined as either underlying rhythm or RVP for those with complete heart block (CHB). For each patient, the *OPM* was defined as the pacing modality that yielded the largest improvement in BiVAT-90. Dyssynchrony metrics for different pacing modalities compared to baseline were tested using paired-sample *t* tests. Statistical analysis was performed using the Stata Statistical Software Package Release 17 (StataCorp LLC, College Station, TX). *P* < .05 was considered significant.

## Results

### Baseline characteristics

Ten patients were recruited, and their characteristics are summarized in Table 1. All patients had previously implanted WiSE-CRT devices as part of the WiSE postmarket registry^6^ or the Stimulation of the Left Ventricular Endocardium for Cardiac Resynchronization Therapy trial.^19^ The mean age was 72.7 ± 9 years; 7 patients were male. Four patients had an ischemic heart failure etiology. The leadless CRT indication was conventional CRT nonresponse in 1 patient, with the remaining 9 being CRT-naive due to failure of LV lead implantation. Five patients had LV septal RE implants, and 5 had LV lateral wall implants. In 2 patients with septal implants, the RE had been deployed at the site of a mapped left bundle (LB) potential. In the remaining 3, implantation was performed without septal mapping. Four patients had underlying SR with left bundle branch block (LBBB). The remaining 6 had atrial fibrillation (AF), 1 with slow ventricular response and 5 with CHB. The protocol was completed in all 10 patients. The total protocol time varied between 15 and 25 minutes.

**Table 1:** Baseline characteristics

Patient no.	Sex	Etiology	WiSE-CRT indication	Underlying rhythm	RV lead location	LV electrode location	Scar location
1	Male	Nonischemic	Failed LV lead	AF, CHB	Apical	Basal inferoseptum	Inferior
2	Female	Nonischemic	Failed LV lead	SR, LBBB	Apical	Mid-septum	N/A
3	Male	Nonischemic	Nonresponder	AF, CHB	Apical	Basal septum	Apical lateral
4	Male	Ischemic	Failed LV lead	SR, LBBB	Apical	Mid-septum	Inferolateral
5	Male	Ischemic	Failed LV lead	AF, CHB	Apical	Basal septum	Septum, inferior wall, apex
6	Female	Nonischemic	Failed LV lead	SR, LBBB	Apical	Posterolateral	N/A
7	Male	Ischemic	Failed LV lead	Slow AF	Apical	Lateral	Lateral
8	Female	Nonischemic	Failed LV lead	SR, LBBB	Apical	Basal lateral	N/A
9	Male	Ischemic	Failed LV lead	AF, CHB	Apical	Mid lateral	Inferior, mid inferolateral
10	Male	Nonischemic	Failed LV lead	AF, CHB	Apical	Apical anterolateral	N/A

AF, atrial fibrillation; BiV = biventricular; BIVAT-90 = time for 90% biventricular activation; BiVDI = biventricular dyssynchrony index; CHB, complete heart block; LBBB, left bundle branch block; LVAT95 = time for 95% left ventricular activation; LVDI = left ventricular dyssynchrony index; RVAT90 = time for 90% right ventricular activation; SR, sinus rhythm.

### Main results

**Figure 1:**
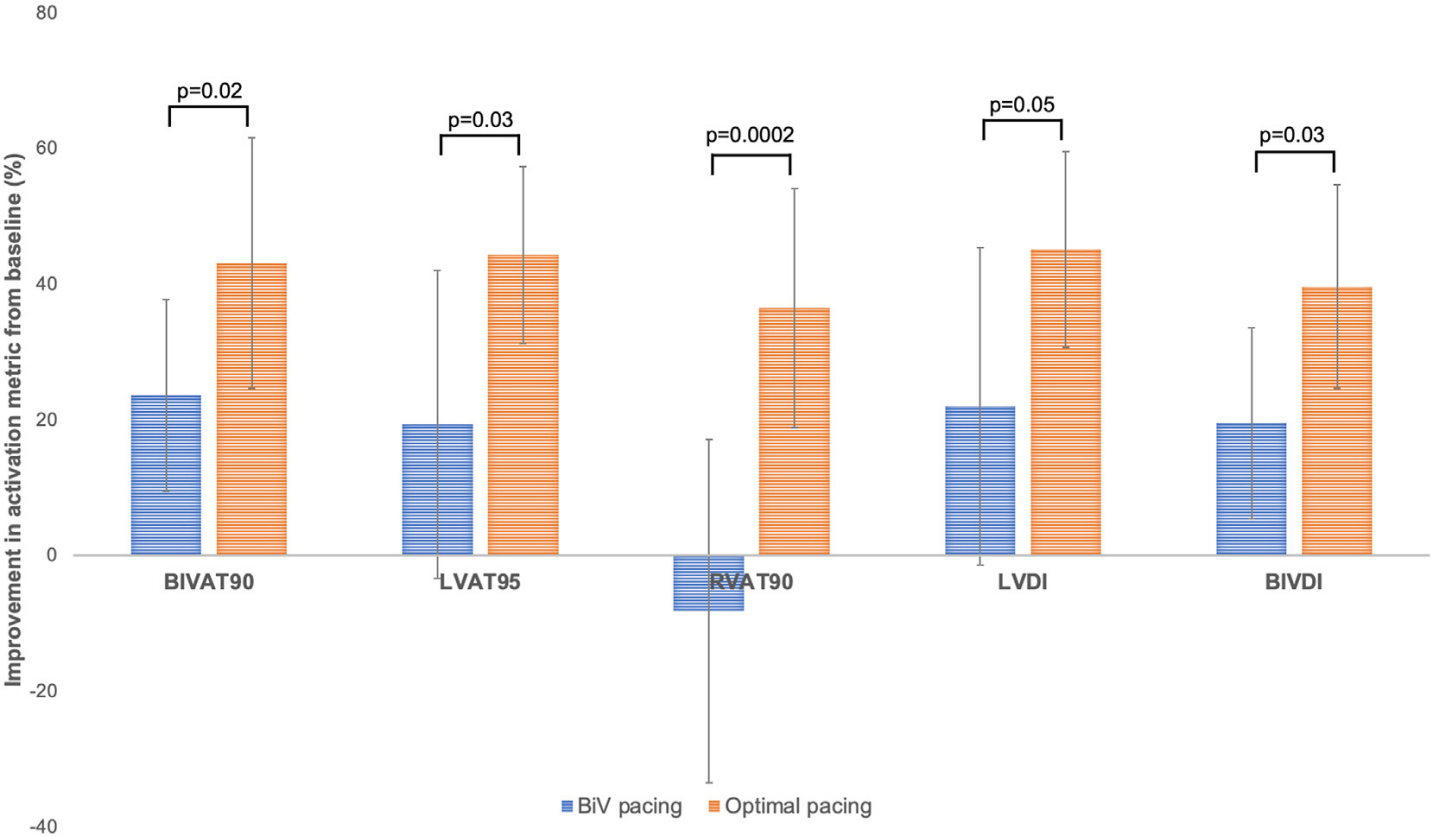
Improvement from baseline (underlying rhythm or RVP for those with CHB) in each activation metric when the optimal pacing modality is chosen (*orange bars*) compared to when empirical BiV pacing is chosen (*blue bars*). BiV = biventricular; BIVAT-90 = time for 90% biventricular activation; BiVDI = biventricular dyssynchrony index; LVAT95 = time for 95% left ventricular activation; LVDI = left ventricular dyssynchrony index; RVAT90 = time for 90% right ventricular activation.

The changes in each activation metric with BiVP and pacing at an individualized OPM compared to baseline are demonstrated in Figure 1. BiVP yielded a 23% ± 18% improvement in BiVAT-90 (*P* < .01) and a 19% ± 18.5% improvement in BiVDI (*P* = .02). Nonsignificant improvements of 22.7% and 23.4% were observed in LVAT-95 and LVDI, respectively, with a nonsignificant prolongation in RVAT-90 of 8.3%. At an individualized OPM, significant improvements are observed in all metrics compared to BiVP. At the OPM, there was a 43% ± 14.1% improvement in BiV activation compared to baseline, which was 20% superior to BiVP (*P* = .02), and a 44% ± 16.3% improvement in LVAT-95, which was 24% superior to BiVP (*P* = .03). The OPM yielded significant reductions in RVAT-90 of 36% ± 25.1%, which was significantly better than the 8% prolongation seen during empirical BiVP (*P* < .01). Using an OPM, BiVAT-90 was significantly improved in both patients with lateral (43.3%; *P* = .0001) and septal (42.4%; *P* = .009) LV implants.

A color-coded illustration of the patient-specific OPM is provided in Table 2. As can be seen, there was considerable interindividual heterogeneity, also demonstrated in Online Supplemental Figure S2, which shows the changes in each activation metric for each pacing modality and displays wide confidence intervals. Based on the considerable interindividual heterogeneity, a subgroup analysis was undertaken for patients with septal RE implants and patients in SR.

**Table 2:** Patient-specific optimal pacing modality for each activation metric

Patient no.	Underlying rhythm	Electrode position	Optimal pacing modality				
			BiVAT-90	LVAT-95	RVAT-90	LVDI	BiVDI
1	AF, CHB	Septal	LV	LV	LV	LV	LV
2	SR, LBBB	Septal	LV	LV-OPT	LV-OPT	LV-OPT	LV-OPT
3	AF, CHB	Septal	BiV	LV	BiV	LV	BiV
4	SR, LBBB	Septal	LV-OPT	LV-OPT	LV-OPT	LV-OPT	LV-OPT
5	AF, CHB	Septal	LV	LV	LV	LV	LV
6	SR, LBBB	Lateral	LV-OPT	BiV	LV-OPT	BiV	LV-OPT
7	AF, CHB	Lateral	BiV	BiV	LV	BiV	BiV
8	SR, LBBB	Lateral	LV-OPT	LV-OPT	LV-OPT	LV-OPT	LV-OPT
9	AF, CHB	Lateral	BiV	BiV	RV	BiV	BiV
10	AF, CHB	Lateral	BiV	BiV	BiV	BiV	BiV

*Yellow* denotes empirical BiV pacing; *green*, empirical LV-only pacing; *blue*, LV-OPT, ie, LV-only pacing at the electrically optimized AV delay; and *red*, RV pacing.

AF, atrial fibrillation; BiV = biventricular; BIVAT-90 = time for 90% biventricular activation; BiVDI = biventricular dyssynchrony index; CHB, complete heart block; LBBB, left bundle branch block; LV, left ventricular; LV-OPT, LVP with an optimized atrioventricular delay; SR, sinus rhythm.

### Subgroup analysis—LV septal pacing

**Figure 2:**
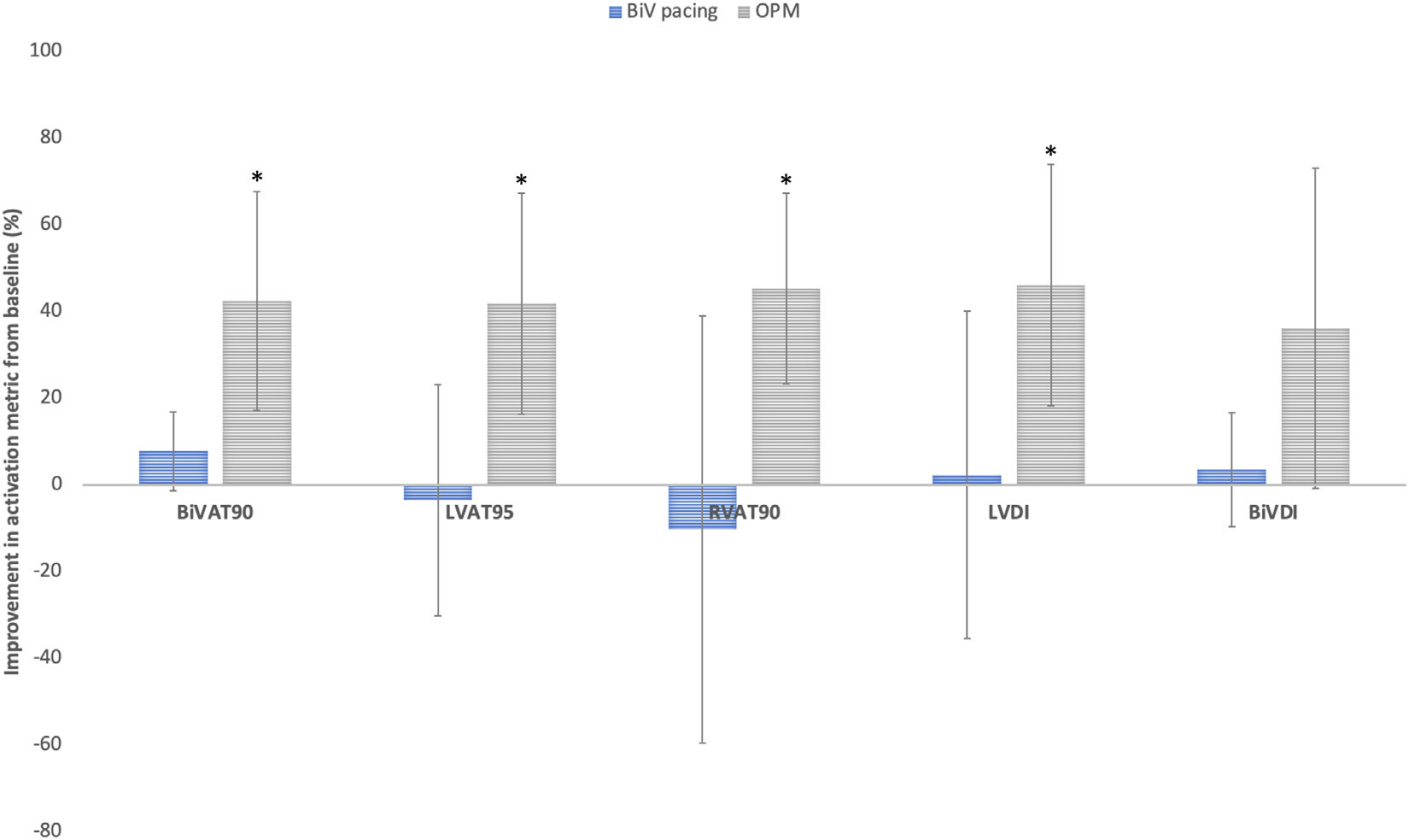
Mean improvement in each activation metric with BiV pacing and at the optimal pacing modality (OPM) compared to baseline (underlying rhythm or RVP for those with CHB)—septal implant subgroup. ∗*P* < .05. BiV = biventricular; BIVAT-90 = time for 90% biventricular activation; BiVDI = biventricular dyssynchrony index; LVAT95 = time for 95% left ventricular activation; LVDI = left ventricular dyssynchrony index; RVAT90 = time for 90% right ventricular activation.

**Figure 3:**
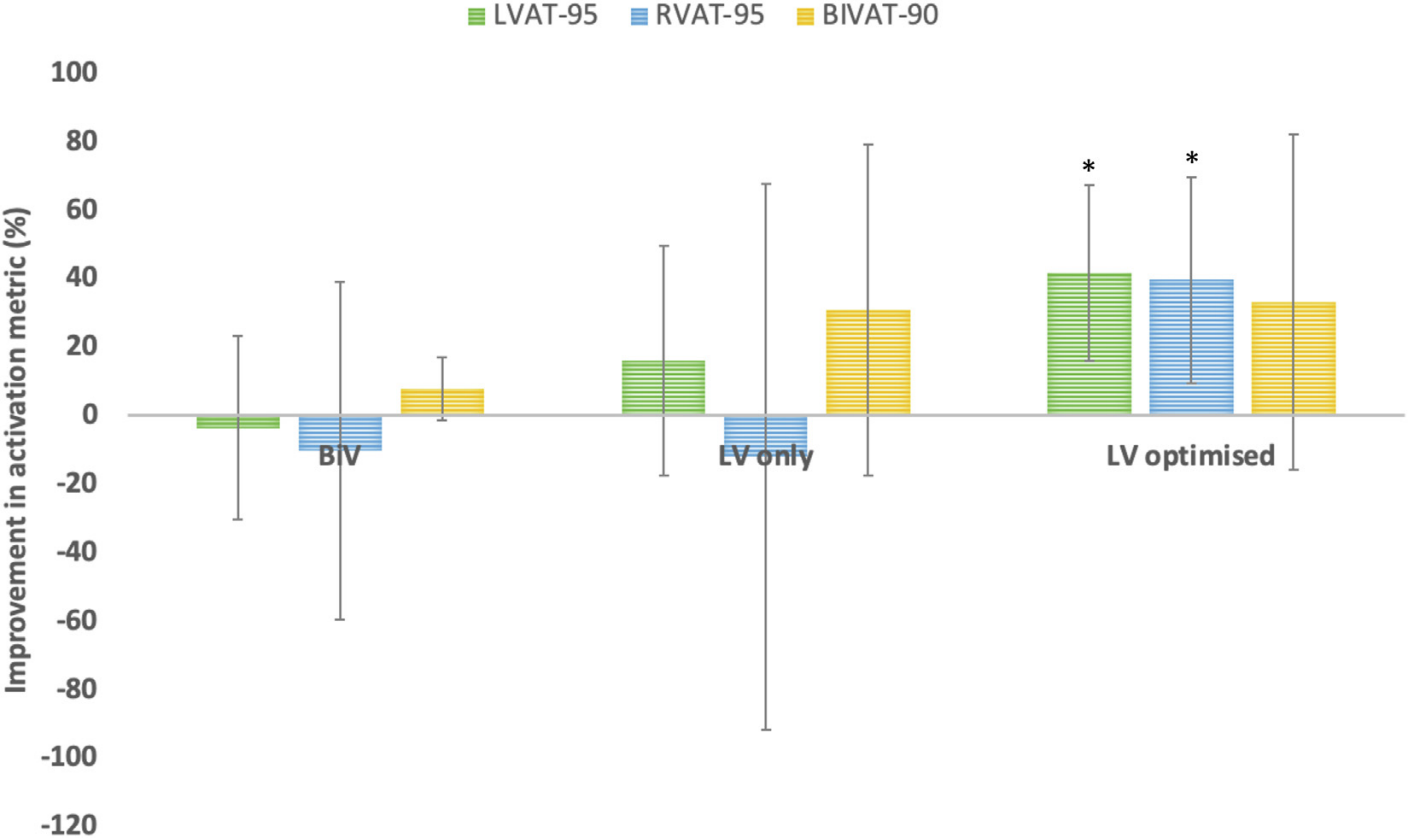
Mean improvement in activation metric with BiV, LV only, and LV only with optimized AV delay compared to baseline (underlying rhythm or RVP for those with CHB)—septal implant subgroup. ∗*P* < .05. AV, atrioventricular; BiV = biventricular; BIVAT-90 = time for 90% biventricular activation; BiVDI = biventricular dyssynchrony index; LV, left ventricular; LVAT95 = time for 95% left ventricular activation; LVDI = left ventricular dyssynchrony index; RVAT90 = time for 90% right ventricular activation.

BiVP in this cohort resulted in a mean improvement in BiVAT-90 of 7.8% ± 7.3% (*P* = .08). At an OPM, there were significant improvements in all activation metrics, in particular a 42% ± 20.4% improvement in BiVAT-90 (*P* < .01) (Figure 2). Three patients had underlying AF; of these patients, LVP was the OPM in 2 and BiVP was the OPM in the other. Of the 2 patients in SR, LV-OPT was the OPM in 1 patient, with empirical LVP performing best in the other. Overall in this cohort, LVP, with or without AVD optimization, outperformed BiVP in 4 of 5 patients. LVP resulted in an improvement in BiVAT-90 of 30.9% compared to baseline, translating to a 23% improvement over BiVP, driven by a reduction in LVAT-95 (Figure 3), but not RVAT-90. LV-OPT led to a 33.1% improvement in BiVAT-90 compared to baseline, 25.5% superior to BiVP, with improvements observed in both LVAT-95 and RVAT-90 (Figure 3).

The ECG features of conduction system capture in these patients are provided in Online Supplemental Table S2. The mean ECG-derived stimulation to LV activation time was 82.4 ± 24 ms. LVP displayed a stimulation to LV activation time of ≤80 ms in 3 of 5 patients including both patients where an LB potential was identified at the deployment site. Examples of both posterior fascicular capture (patient 2) and more proximal LB capture (patient 3) are shown in Online Supplemental Figure S3.

### Subgroup analysis—SR

**Figure 4:**
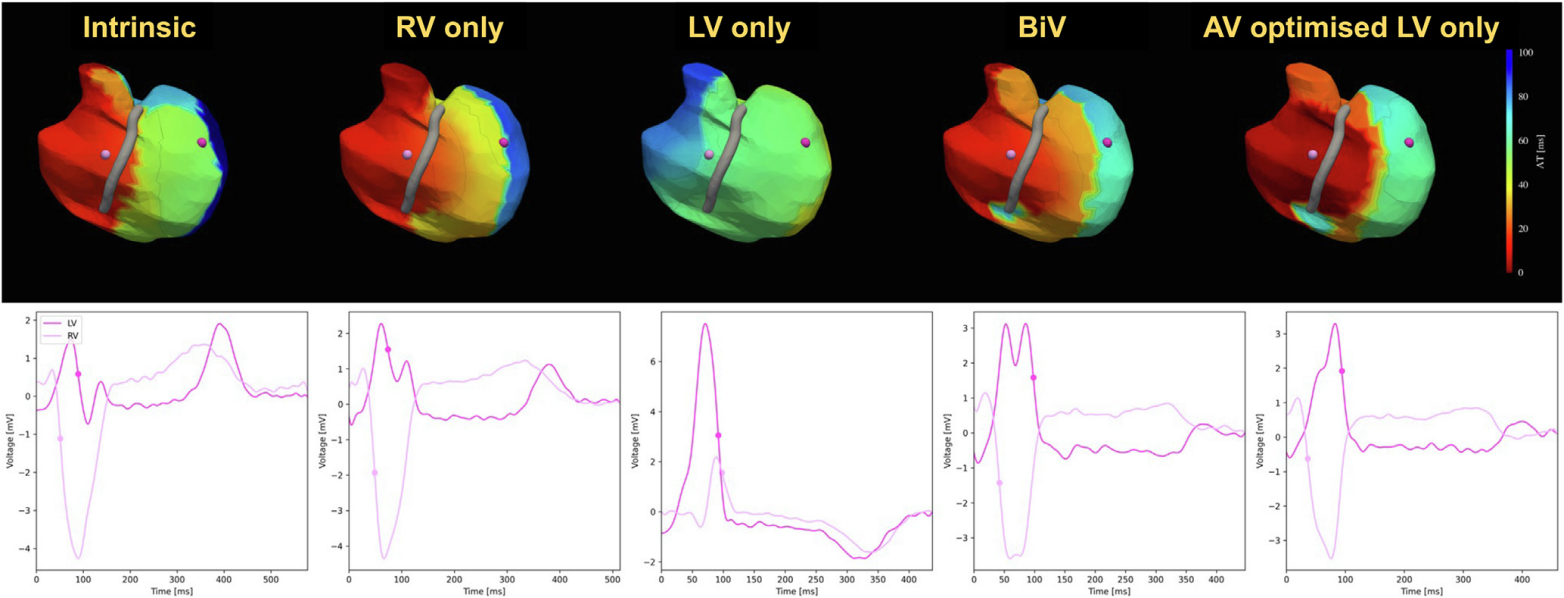
Activation maps for patient 2 (septal implant) at each pacing modality. *Top row:* Activation time maps. The *red and blue areas* indicate early and late activation. *Bottom row:* Unipolar electrograms for 1 LV and 1 RV location are indicated in *pink* on the activation maps above. The *dots* on the electrograms indicate the annotated activation times. BiV = biventricular; BIVAT-90 = time for 90% biventricular activation; BiVDI = biventricular dyssynchrony index; LV, left ventricular; LVAT95 = time for 95% left ventricular activation; LVDI = left ventricular dyssynchrony index; RVAT90 = time for 90% right ventricular activation.

**Figure 5:**
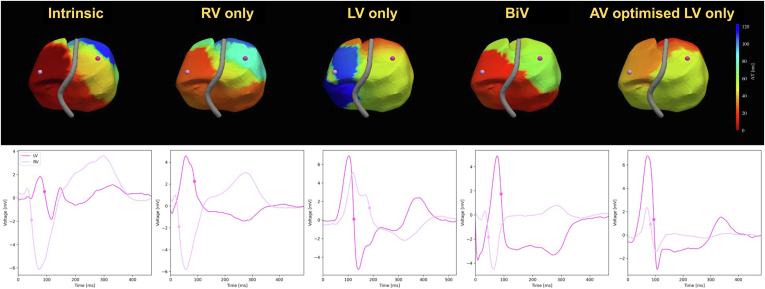
Activation maps (*top row*) and unipolar electrograms for 1 LV and 1 RV location (*bottom row*) for patient 8 (lateral LV implant) at each pacing modality. BiV = biventricular; BIVAT-90 = time for 90% biventricular activation; BiVDI = biventricular dyssynchrony index; LV, left ventricular; LVAT95 = time for 95% left ventricular activation; LVDI = left ventricular dyssynchrony index; RV, right ventricular; RVAT90 = time for 90% right ventricular activation.

Four patients were in SR at the time of the study (2 with septal implants and 2 with lateral implants). These patients exhibited a 44.5% ± 6.2% improvement in BiVAT-90 with LV-OPT compared to baseline (*P* < .01). This translated to a 23% improvement compared to empirical BiVP (*P* = .08). VOO LVP did not show any significant differences in BiVAT-90 from baseline in this cohort (−5% ± 51%; *P* = .85), driven by long RV activation times in patients with LV lateral implants. The epicardial activation map patterns showed that LVP led to delayed RV activation in patients with both lateral and septal LV implants, where the basal RV activated late. The maps demonstrate that the benefit of LV-OPT for patients with SR and LBBB is from fusion of the LV paced impulse with intrinsic right bundle branch (RBB) conduction (Figure 4, septal implant, and Figure 5, lateral implant). This is quantified by a significant improvement in RVAT-90 with LV-OPT (52.8% ± 20.2%) compared to LVP (−106.7% ± 51.5%; *P* < .01) or BiVP (−14.9% ± 34%; *P* < .01).

**Figure 6:**
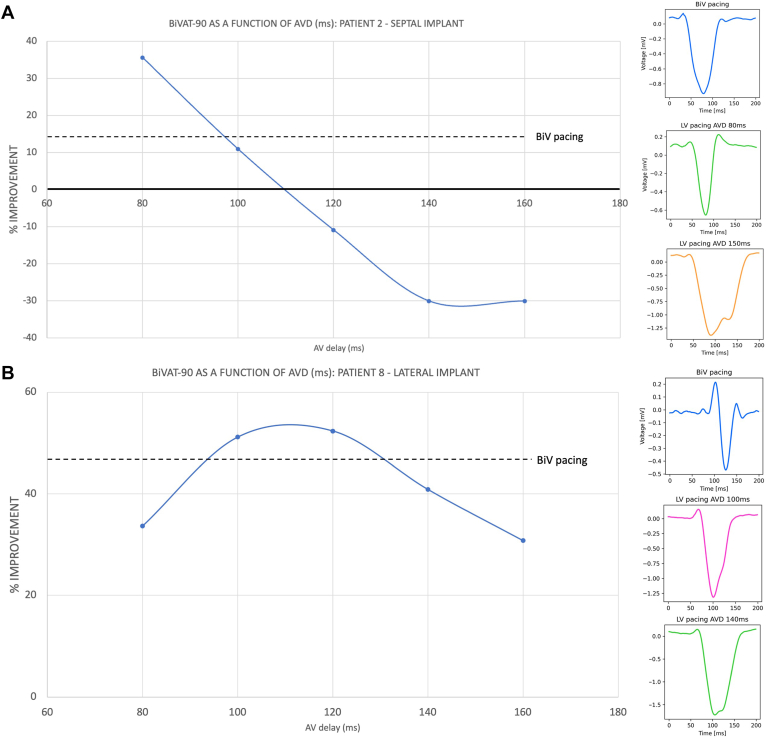
Improvement in BiVAT-90 at varying AV delays (AVDs) using synchronous LV-only pacing compared to baseline, with example ECGi-derived lead V_1_ equivalent electrograms (*right panels*). BiVAT-90 improvement using BiV pacing is demonstrated with the *dashed line*. **A:** Patient 2—septal implant. **B:** Patient 8—lateral implant. AV, atrioventricular; BiV = biventricular; BIVAT-90 = time for 90% biventricular activation; BiVDI = biventricular dyssynchrony index; ECGi, electrocardiographic imaging; LV, left ventricular; LVAT95 = time for 95% left ventricular activation; LVDI = left ventricular dyssynchrony index; RVAT90 = time for 90% right ventricular activation.

Figure 6A demonstrates BiVAT-90 as a function of AVD using LVP with the optimal delay generating a 35% improvement from baseline and 20% improvement compared to BiVP, with a steep drop-off at nonoptimal delays. By contrast, in a patient with a lateral LV implant, the optimal delay yielded only a 5% improvement over BiVP, with modest drop-offs at nonoptimal delays (Figure 6B).

## Discussion

This was the first study to perform electroanatomical characterization of leadless CRT and the first to evaluate how treatment efficacy could be optimized. The findings were as follows:

1. Biventricular leadless endocardial CRT improves ECGi-derived electrical activation and dyssynchrony metrics.
2. Leadless LV septal endocardial pacing improves ECGi-derived metrics at the OPM.
3. BiVP may not be the OPM in all patients. The OPM is dependent on patient factors, such as the underlying rhythm, and procedural factors, such as the location of the RE implant.
4. For those receiving leadless LV septal pacing, LVP may be superior to BiVP.
5. For those with SR and LBBB, AV-optimized LVP may be superior to BiVP, particularly in patients with septal implants.

### Efficacy of leadless CRT

Our findings are consistent with previous studies examining the effects of *temporary* LV endocardial pacing on ECGi-derived activation. Optimal pacing improved BiVAT-90 by >40% in the overall study population and in the septal implant subgroup. This is comparable to findings from Elliott et al,^17^ who reported that both temporary BiV endocardial pacing (lateral wall LV stimulation) and temporary left bundle branch area pacing (LBBAP; LV septal stimulation) generated an improvement in BiVAT-90 of ∼50%, with a 15% improvement in acute hemodynamic response. In that study, BiV epicardial CRT resulted in less pronounced improvements over baseline (33%) compared to BiV endocardial pacing or LBBAP. Studies have reported that LV endocardial pacing may outperform epicardial pacing because of the higher conduction velocity of endocardial tissue and the unrestricted pacing locations allowing operators to avoid myocardial scar.^20^, ^21^, ^22^ However, uptake of permanent transvenous endocardial pacing systems is limited by prohibitively high stroke rates.^23^ Our study shows for the first time that endocardial CRT delivered through a *permanent* leadless system can generate a similar degree of electrical activation and dyssynchrony improvement observed during temporary LV endocardial pacing. This suggests that leadless technology allows the benefits of endocardial pacing to be safely harnessed in a real-world setting.

### Fusion pacing in leadless CRT

Fusion pacing refers to CRT delivery programmed to preserve intrinsic AV conduction via the RBB in patients with underlying LBBB.^24^ While long-term outcome data are awaited, studies using conventional CRT have demonstrated improvements in ECG and ECGi-derived metrics of electrical dyssynchrony^25^^,^^26^ and acute hemodynamic benefits.^27^ LVP from a coronary sinus branch has also been shown to generate similar improvements in QRS duration to conventional BiVP when a rate-adaptive AVD algorithm to promote fusion is applied.^28^ Our study is the first to describe a similar potential utility of leadless endocardial pacing to deliver LV-only fusion pacing, which may provide superior resynchronization to BiVP in selected patients.

### Leadless LV septal pacing

Epicardial activation mapping suggests that LV septal endocardial pacing improves BiV and LV activation compared to baseline; however, RV activation appeared delayed. This is consistent with previous studies examining transvenous LBBAP using ECGi^29^ and ultra-high-frequency ECG,^15^ which describe delayed RV activation with nonselective LBB capture. In our cohort, septal LVP led to improvements in BiVAT-90 predominantly through improvements in LVAT-95. However, superior results were observed in patients in SR when the optimal AVD was set, with significant improvements in RVAT-90. This suggests that fusion pacing may be important in achieving the best possible results from LV septal endocardial pacing supporting prior in silico^30^ and in vivo^31^ evidence demonstrating the benefits of AV optimization with transvenous LBBAP. Indeed, our results indicate that BiV activation is particularly sensitive to AVD modification in those receiving LV septal compared to lateral wall pacing. The mechanism of this phenomenon is not clear. We postulate, however, that at nonoptimal delays, an LV septal paced wavefront may block intrinsic RBB conduction. Similarly, RV apical pacing may cause distal His-Purkinje tissue block, thereby inhibiting the propagation of the LV septal paced wavefront during BiVP. This may explain why BiVP was less favorable than LVP in this study subgroup. Our results thus suggest that while leadless LV septal pacing shows promise, further study into how to clinically deliver LVP may be needed to maximize this potential.

### Future directions in leadless CRT and leadless CSP

The theoretical benefits of LV endocardial septal pacing stem from the potential to achieve left-sided conduction system capture.^14^^,^^32^^,^^33^ In our cohort of study patients, intraprocedural end points for CSP^34^ were not targeted; however, ECG features of CSP were nevertheless observed in 3 patients. The next stage of research would be evaluation of outcomes when leadless pacing is performed to meet target parameters such as implantation at the site of an LBB potential or demonstration of selective and nonselective capture. The upcoming Achieving Conduction System Activation With Leadless Left Ventricular Endocardial Pacing trial (ClinicalTrials.gov identifier NCT05659680) aims to prospectively evaluate the feasibility of leadless CSP. A further area requiring attention is the delivery of LVP through a leadless device, as our findings suggest that this may be beneficial not only in patients with septal implants but possibly also in patients with lateral implants via fusion pacing. Current leadless devices used for RVP, such as Micra (Medtronic), are too large to safely pace the LV without adverse effects.^35^ As technology becomes more compact, the future will likely see “stand-alone” leadless LVP devices without the requirement of a coimplant. Of note, the WiSE-CRT device could potentially deliver LVP by timing its impulse at a fixed interval after an *atrial* pacing event rather than an *RVP* event. This technique has yet to be demonstrated in humans; however, testing its feasibility may be useful in optimizing leadless CRT using currently available technology.

### Limitations

This is a mechanistic study with a small sample size and heterogeneous patient and procedure characteristics. This is representative of a real-world cohort of patients with advanced heart failure who receive these devices and have a high incidence of characteristics such as AF, CHB, and ischemic cardiomyopathy. The sample size also reflects the novelty of the technology being evaluated. In particular, leadless LV septal pacing has been performed in only 8 patients worldwide, 5 of whom are recruited into this study. As such, at this time it was not possible to implement more stringent inclusion criteria to homogenize the study population, for example, the position of the LV electrodes. These had been previously implanted, with the LV implant location chosen at the operator’s discretion based on delivery sheath stability and thresholds. In addition, it was not possible to recruit a larger pool of patients in SR at the time of testing. As such, subgroup patient numbers were too small to make statistical comparisons between BiVP, LVP, or LV-OPT. However, by defining the OPM, we were able to demonstrate to a statistically significant degree that leadless CRT improves electrical activation compared to baseline, if device settings are optimized on an individualized basis. In this regard, our findings should be considered hypothesis generating and open avenues of investigation into which patients will likely benefit from certain LV implant locations or device settings.

Regarding ECGi mapping, this has the advantage of being quick and noninvasive. Its disadvantage is being unable to directly visualize the septum, as the maps generated are the sites of epicardial breakout. Invasive mapping of the septum would be useful in fully characterizing modalities such as BiV LV septal pacing and fusion pacing, and this is one of the aims of the upcoming Achieving Conduction System Activation With Leadless Left Ventricular Endocardial Pacing study.

## Conclusion

This is the first study to characterize the electroanatomical effects of BiV endocardial pacing and LV septal pacing using a permanent system. Our findings demonstrate the acute electrical efficacy of leadless CRT and leadless LV septal pacing as CRT modalities. While current recipients of this technology are a niche cohort of patients with heart failure, the areas of interest highlighted in this study are applicable moving forward as the field of leadless pacing in general continues to evolve and expand.

Developments in pacing techniques and technologies are vital to unlocking the potential of leadless CRT and leadless CSP, with individual patient optimization likely being the key to maximizing system performance.
