# Supplementary material for: Optimizing electrical efficacy of leadless cardiac resynchronization therapy and leadless left ventricular septal pacing: Insights on left and right ventricular activation from electrocardiographic imaging
Source: Heart Rhythm O2. 2024 Jul 5;5(8):551–60. doi: 10.1016/j.hroo.2024.07.004 (PMC11385406; doi:10.1016/j.hroo.2024.07.004)
Supplement: Supplemental Tables and Figures [file mmc1.docx]

**Supplementary Material – Optimization of Leadless CRT**

**Supplemental ECGi methodology**

For each pacing configuration recording (repeated three times), one good quality beat was isolated within the final 10 seconds of the recording and exported to analyse offline. The ventricular electrograms were band-pass filtered between 0.5 and 80 Hz to reduce noise, based on previous ECGi studies.(1) A window to isolate the QRS segment was defined manually for activation time computation. Activation time for each ventricular point was defined as the time of maximal negative derivative (dV/dtmax) of the QRS segment of the unipolar electrogram. For low amplitude electrograms (amplitude within the QRS segment < 0.5, activation time cannot be reliably calculated due to leftover noise after filtering.(1) To avoid including these poor-quality signals in the analysis, we automatically excluded points with low amplitude. We also analysed the statistical distribution of the resulting activation times and removed outliers (<1st quartile – [1.5 × interquartile range (IQR)]; or >3rd quartile + [1.5 × IQR]) from such distribution, assuming that an activation time annotation outside these ranges indicated a wrong annotation due to noise. Due to the high number of electrograms per patient (>2000) and to the number of analysed beats, manually reviewing all the electrograms was not feasible. Therefore, this protocol was applied automatically to all analysed beats. For all beats for all patients, this resulted in excluding a maximum of less than 2% of the ventricular electrograms (in most cases between 0 and 1%), indicating that most electrograms were of sufficiently good quality to reliably compute the activation time.

Poor-quality electrograms were thus automatically removed and interpolated assuming zero uncertainty on the known annotated activation times.(2)

The outflow tracts and peri-annular regions were excluded. This produced a ventricular activation map where each point is annotated with an activation time for each pacing configuration.

**
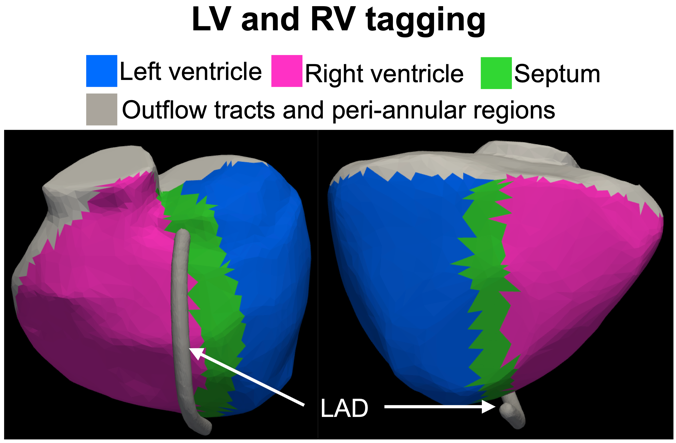
**

**ECGi validation**

Validation of our ECGi-derived metrics for this cohort was performed by correlating baseline QRSd with BiVAT-90. This showed a strongly positive correlation with a correlation coefficient of 0.803 (p<0.01), see Supplementary Figure S1.

Different groups have assessed ECGi accuracy in replicating activation patterns and dyssynchrony recorded using invasive systems. Bear et al compared ECGi measurements with a 108-electrode sock in perfused pig hearts, showing that ECGi can replicate the invasively recorded activation patterns during intrinsic rhythm.(3) Similarly, in another study, Bear et al compared interventricular dyssynchrony between the same two systems in 11 perfused pig hearts with induced LBBB during intrinsic rhythm, biventricular pacing, RV pacing and LV pacing.(4) These results showed that ECGi was able to accurately detect different levels of dyssynchrony. Graham et al compared ECGi and CARTO contact mapping recordings in 8 patients, again showing consistency between the activation pattern obtained with the two recording systems.(1) In conjunction with our own ECG-based validation, these studies provide evidence that the ECGi system is able to accurately detect dyssynchrony and to replicate invasively recorded activation patterns, increasing confidence in the results presented in this study.

**SUPPLEMENTARY TABLES AND FIGURES**

**Supplementary Table S1** – Device settings used in temporary pacing protocol.

| **Study pacing modality** | **Acronym** | **Device settings** |
| --- | --- | --- |
| RV pacing | RVP | VOO through co-implant with WiSE-CRT device deactivated. |
| BiV pacing | BiVP | Clinically programmable setting for WiSE-CRT, whereby LV electrode detects RV pacing pulse, and paces near simultaneously (3-5ms delay). Standard device output (2 x threshold level). |
| LV only pacing | LVP | VOO through the WiSE-CRT programmer. Co-implant set at VVI 40 bpm. Standard device output (2 x threshold level). QRS morphology monitored during threshold test to assess for possible non-selective-selective capture transition. |
| LV only pacing at optimised AV delay. AV delays from 80-200ms tested. | LV-OPT | DOO via the co-implanted device with the RV lead set to deliver a subthreshold impulse, which therefore does not capture myocardium. The WiSE-CRT device is set to pace in response to detection of the RV pacing impulse, thus resulting in LV only pacing at the set paced AV delay. Standard device output (2 x threshold level). |

**Supplementary Table S2** – ECG features of conduction system pacing in patients with LV septal implants.

| **Patient** | **LV electrode location** | **Implant site LB potential?** | **V1 morphology** | **Stim-LVAT (ms)** | **Non-selective to selective capture transition?** |
| --- | --- | --- | --- | --- | --- |
| 1 | Basal inferoseptum | N | qRs | 120 | N |
| 2 | Mid-Septum | Y | Rs | 60 | N |
| 3 | Basal Septum | N | rSR’ | 80 | N |
| 4 | Mid-Septum | Y | qRS | 64 | N |
| 5 | Basal Septum | N | Rs | 88 | N |

**Supplementary Figure S1** – Correlation between 12 lead ECG baseline QRSd and BiVAT-90.

**Supplementary Figure S2 -** Improvement from baseline (underlying rhythm or RVP for those in CHB) in each activation metric, for each tested pacing modality – whole cohort

**
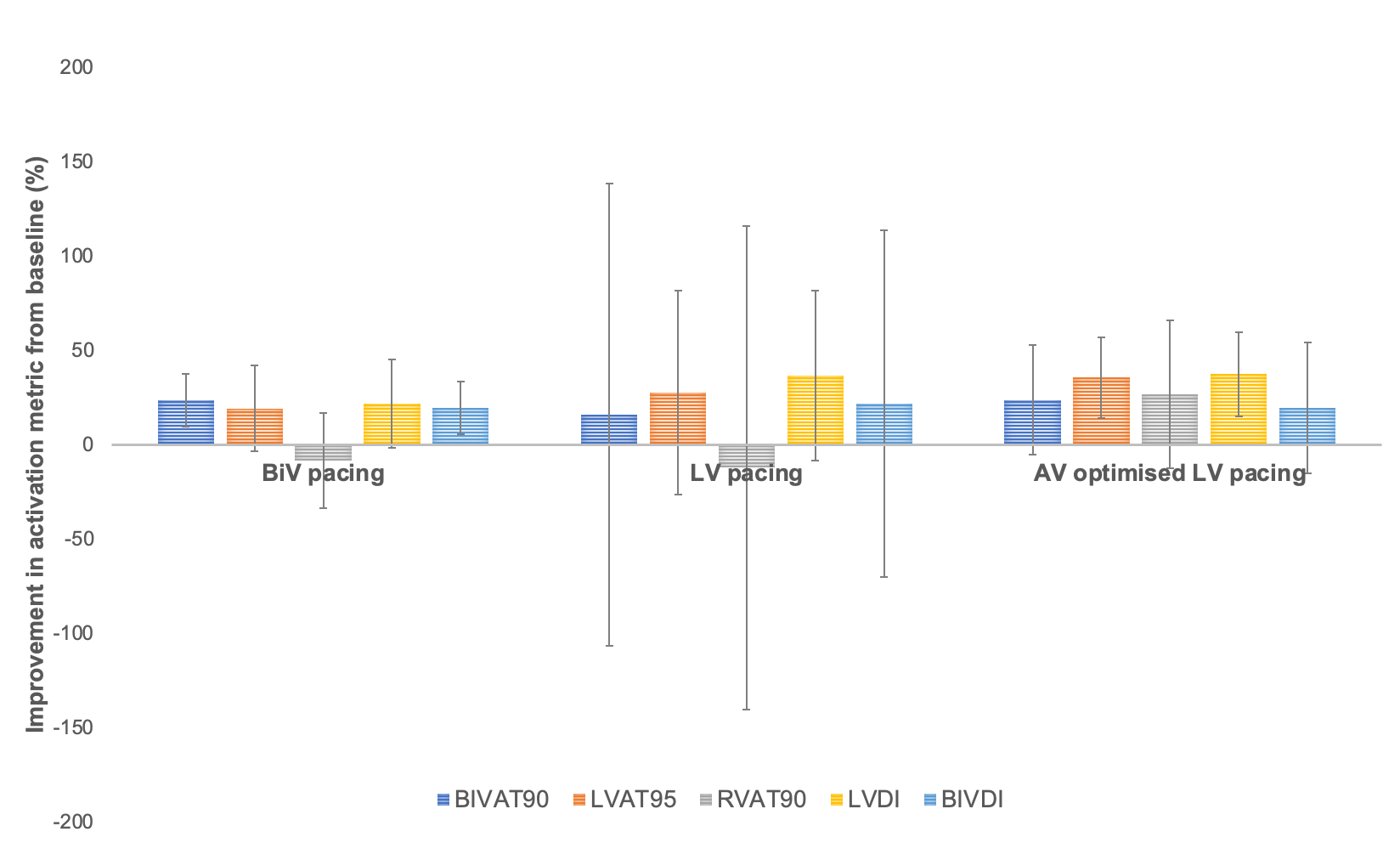
**

**Supplementary Figure S3** – 12-lead ECG of LV only pacing for patient 2 (A) and patient 3 (B). In patient 2, whose electrode was placed in the mid septum, there is evidence of possible left posterior fascicular capture (leftward axis). In patient 3, whose electrode was more basal, the axis may suggest more proximal left bundle area capture.

**Supplementary References**

1. Graham AJ, Orini M, Zacur E, Dhillon G, Daw H, Srinivasan NT, et al. Simultaneous Comparison of Electrocardiographic Imaging and Epicardial Contact Mapping in Structural Heart Disease. Circ Arrhythm Electrophysiol. 2019 Apr;12(4).

2. Coveney S, Corrado C, Roney CH, O’Hare D, Williams SE, O’Neill MD, et al. Gaussian process manifold interpolation for probabilistic atrial activation maps and uncertain conduction velocity. Philosophical Transactions of the Royal Society A. 2020 Jun 12;378(2173).

3. Bear LR, Bouhamama O, Cluitmans M, Duchateau J, Walton RD, Abell E, et al. Advantages and pitfalls of noninvasive electrocardiographic imaging. J Electrocardiol. 2019 Nov 1;57:S15–20.

4. Bear LR, Huntjens PR, Walton RD, Bernus O, Coronel R, Dubois R. Cardiac electrical dyssynchrony is accurately detected by noninvasive electrocardiographic imaging. Heart Rhythm. 2018 Jul 1;15(7):1058–69.
